# The genome sequence of the Eurasian river otter, Lutra lutra Linnaeus 1758

**DOI:** 10.12688/wellcomeopenres.15722.1

**Published:** 2020-02-19

**Authors:** Dan Mead, Frank Hailer, Elisabeth Chadwick, Roberto Portela Miguez, Michelle Smith, Craig Corton, Karen Oliver, Jason Skelton, Emma Betteridge, Jale Doulcan Doulcan, Olga Dudchenko, Arina Omer, David Weisz, Erez Lieberman Aiden, Shane McCarthy, Kerstin Howe, Ying Sims, James Torrance, Alan Tracey, Richard Challis, Richard Durbin, Mark Blaxter

**Affiliations:** 1 Wellcome Genome Campus, Wellcome Sanger Institute,, Hinxton, CB10 1SA, UK; 2 Cardiff Otter Project, Cardiff University School of Biosciences, Cardiff, CF10 3AX, UK; 3 Department of Life Sciences, Natural History Museum, London, SW7 5BD, UK; 4 Baylor College of Medicine, Houston, Texas, TX 77030 USA, USA

**Keywords:** Lutra lutra river otter genome sequence chromosomal

## Abstract

We present a genome assembly from an individual male *Lutra lutra* (the Eurasian river otter; Vertebrata; Mammalia; Eutheria; Carnivora; Mustelidae). The genome sequence is 2.44 gigabases in span. The majority of the assembly is scaffolded into 20 chromosomal pseudomolecules, with both X and Y sex chromosomes assembled.

## Species taxonomy

Eukaryota; Metazoa; Chordata; Craniata; Vertebrata; Euteleostomi; Mammalia; Eutheria; Laurasiatheria; Carnivora; Caniformia; Mustelidae; Lutrinae; Lutra; *Lutra lutra* Linnaeus 1758 (NCBI txid 9657).

## Background

The Eurasian river otter, *Lutra lutra*, is found along the coasts and inland waters of Europe, Asia, China, Japan, Java, Sri lanka, the Middle East and North Africa. Eurasia. Throughout Europe, populations of *L. lutra* declined precipitously through the latter half of the 20th century, and the species is of active conservation concern. In Ireland, *L. lutra* populations have remained relatively stable ^1^, and in Britain river restoration and active intervention have resulted in increased populations, and recolonisation of watersheds from which otters had been eliminated ^2^. There is active research of the continuing impacts of pollutants on otters ( Pountney *et al.,* 2015), and on the population genetic patterns that have resulted from their near-extinction and subsequent recovery in Britain ( Stanton *et al.,* 2014). Here we present a chromosomally assembled genome sequence for *L. lutra*, based on a male specimen from Britain.

## Genome sequence report

**Figure 1.**
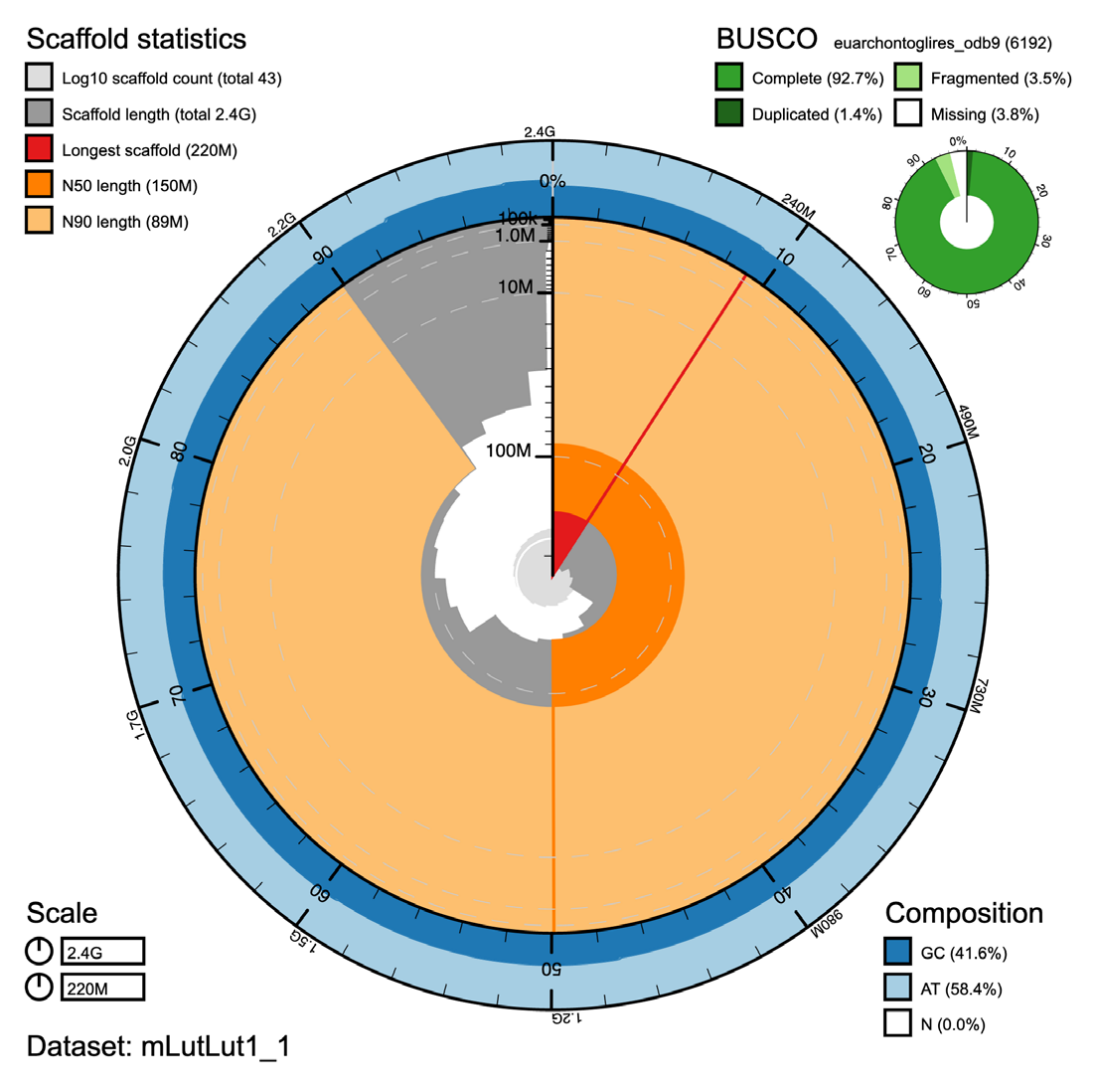
Genome assembly of *Lutra lutra* mLutLut1: BlobToolKit Snailplot. The plot shows N50 metrics for *L. lutra* assembly mLutLut1 and BUSCO scores for the Euarchontoglires set of orthologues. The interactive version of this figure is hosted here.

The genome was sequenced from a naturally deceased single male *L. lutra* collected by the Cardiff Otter Project from Wincanton, Somerset. A total of 63-fold coverage in Pacific Biosciences single-molecule long reads (N50 24 kb) and 58-fold coverage in 10X Genomics read clouds (from molecules with an estimated N50 of 57 kb) were generated. Primary assembly contigs were scaffolded with chromosome conformation HiC data (17-fold coverage). The final assembly has a total length of 2.44 Gb in 43 sequence scaffolds with a scaffold N50 of 149.0 Mb ( Table 1). The majority, 92.7%, of the assembly sequence was assigned to 20 chromosomal-level scaffolds representing 18 autosomes (numbered by sequence length), and the X and Y sex chromosomes ( Figure 1– Figure 4; Table 2). The assembly has a BUSCO ( Simão *et al.,* 2015) completeness of 95.8% using the mammalia_odb9 reference set. While not fully phased, the assembly deposited is of one haplotype. Contigs corresponding to the second haplotype have also been deposited.

**Table 1.** Genome data for *Lutra lutra* mLutLut1.

Project accession data	
Assembly identifier	mLutLut1
Species	*Lutra lutra*
Specimen	NHMUK ZD 2019.215
NCBI taxonomy ID	9657
BioProject	PRJEB35340
Biosample ID	SAMEA994731
Isolate information	Wild casualty; male
Raw data accessions	
PacificBiosciences SEQUEL I	ERR3313238, ERR3313239-ERR3313241, ERR3313246, ERR3313327, ERR3313330, ERR3313333-ERR3313341
10X Genomics Illumina	ERR3316145-ERR3316148, ERR3316169-ERR3316171
Hi-C Illumina	SRR10119468
Genome assembly	
Assembly accession	GCA_902655055.1
*Accession of alternate* *haplotype*	GCA_902653095.1
Span (Mb)	2,438.00
Number of contigs	228
Contig N50 length (Mb)	30.40
Number of scaffolds	43
Scaffold N50 length (Mb)	149.00
Longest scaffold (Mb)	223.45
BUSCO * genome score	C:95.8%[S:94.3%,D:1.5%],F:1.9%,M:2.3%,n:4104

* BUSCO scores based on the mammalia_odb9 BUSCO set using v3.0.2. C= complete [S= single copy, D=duplicated], F=fragmented, M=missing, n=number of orthologues in comparison. A full set of BUSCO scores is available at https://blobtoolkit.genomehubs.org/view/mLutLut1_1/dataset/mLutLut1_1/busco.

**Figure 2.**
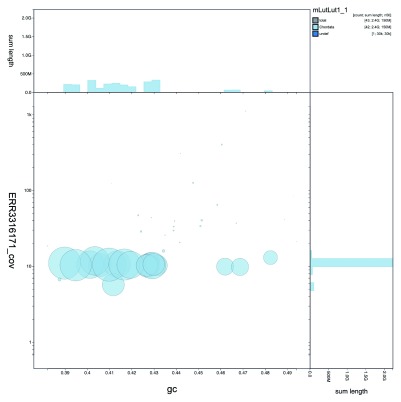
>Genome assembly of *Lutra lutra* mLutLut1: BlobToolKit GC-coverage plot. The interactive version of this figure is hosted here.

**Figure 3.**
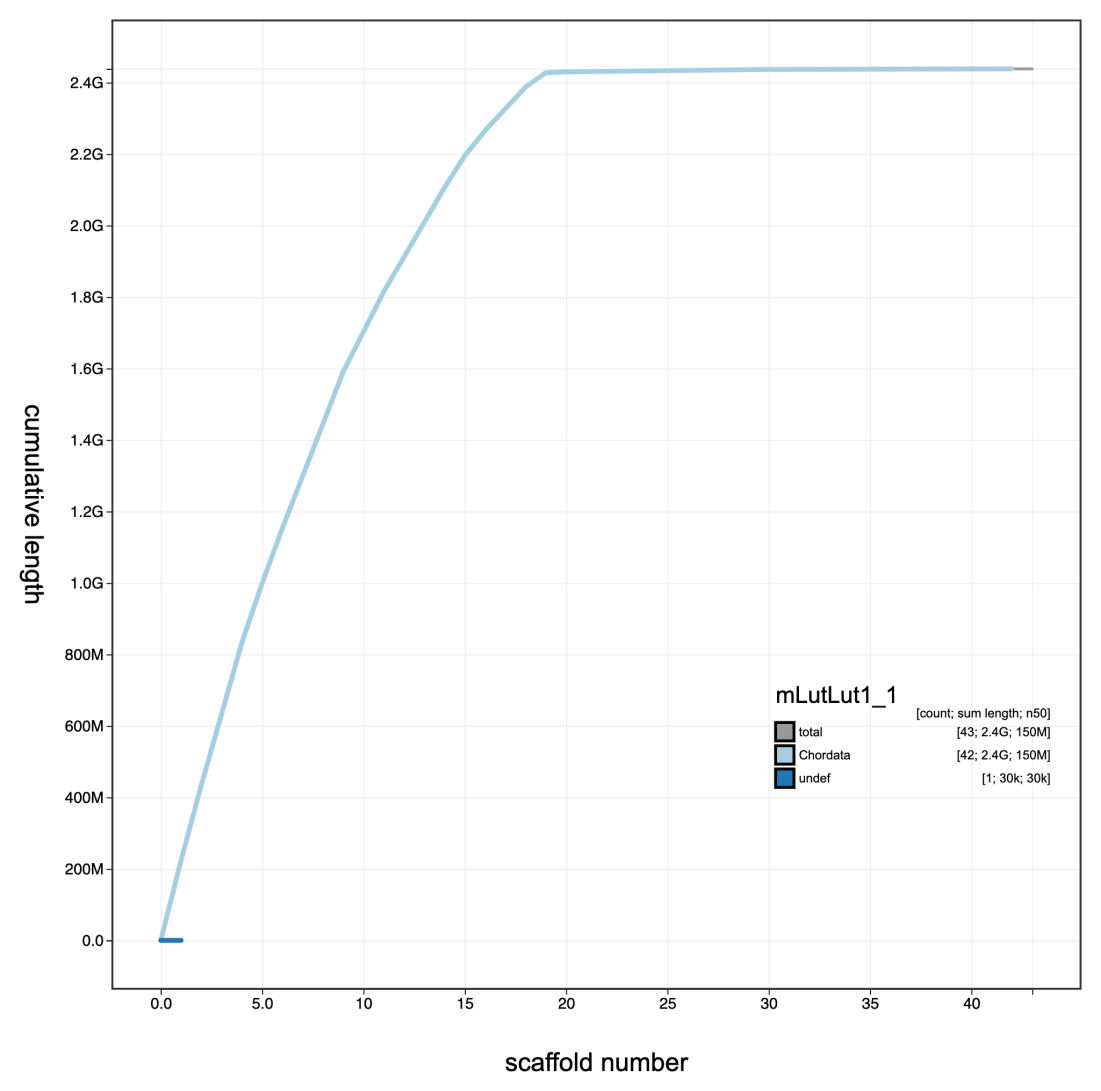
Genome assembly of *Lutra lutra* mLutLut1: BlobToolKit Cumulative sequence plot. The interactive version of this figure is hosted here.

**Figure 4.**
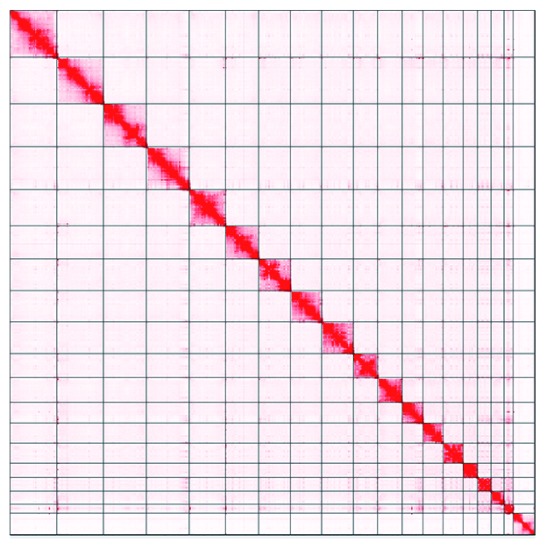
Genome assembly of *Lutra lutra* mLutLut1: Hi-C contact map. Hi-C contact map of the L. lutra mLutLut1 assembly, visualized in Juicebox ( Durand *et al.*, 2016). An interactive version of the map hosted here, powered by Juicebox.js ( Robinson *et al.*, 2018).

**Table 2.** Chromosomal pseudomolecules in the genome assembly of *Lutra lutra* mLutLut1.

*ENA accession*	*Chromosome*	*Size (Mb)*	*GC%*
LR738403.1	1	223.45	41
LR738404.1	2	210.65	39
LR738405.1	3	201.32	39.5
LR738406.1	4	197.71	41.7
LR738407.1	5	165.81	40.3
LR738408.1	6	154.43	40.1
LR738409.1	7	149.01	41.9
LR738410.1	8	144.75	41.3
LR738411.1	9	144.09	42.9
LR738412.1	10	114.66	42.7
LR738413.1	11	108.79	40.6
LR738414.1	12	96.45	43
LR738415.1	13	95.73	42.7
LR738416.1	14	89.08	43.1
LR738417.1	15	69.99	42.8
LR738418.1	16	61.48	46.9
LR738419.1	17	60.35	46.2
LR738420.1	18	40.43	48.2
LR738421.1	X	99.69	41.2
LR738422.1	Y	2.25	38.8

**Table 3.** Software tools used.

Software tool	Version	Source
Falcon-unzip	falcon-kit 1.2.2	( Chin *et al.,* 2016)
purge_dups	1.0.0	( Guan *et al.,* 2020)
3D-DNA	180419	( Dudchenko *et al.,* 2018)
scaff10x	4.2	https://github.com/wtsi-hpag/Scaff10X
arrow	GenomicConsensus 2.3.3	https://github.com/PacificBiosciences/GenomicConsensus
longranger align	2.2.2	https://support.10xgenomics.com/genome-exome/software/ pipelines/latest/advanced/other-pipelines
freebayes	v1.1.0-3-g961e5f3	( Garrison & Marth, 2012)
bcftools consensus	1.9	http://samtools.github.io/bcftools/bcftools.html
gEVAL	2016	( Chow *et al.,* 2016)
BlobToolKit	1	( Challis *et al.,* 2019)

## Methods

The river otter specimen was collected from Wincanton, Somerset by the Cardiff Otter Project. A full tissue dissection and preservation in 80% ethanol was undertaken and the specimen accessioned by the Natural History Museum, London.

DNA was extracted using an agarose plug extraction from spleen tissue following the Bionano Prep Animal Tissue DNA Isolation Soft Tissue Protocol. Pacific Biosciences CLR long read and 10X Genomics read cloud sequencing libraries were constructed according to the manufacturers’ instructions. Sequencing was performed by the Scientific Operations core at the Wellcome Sanger Institute on Pacific Biosciences SEQUEL I and Illumina HiSeq X instruments. Hi-C data were generated by the Aiden lab using an optimised version of their protocols ( Dudchenko *et al.,* 2017).

Assembly was carried out using Falcon-unzip ( Chin *et al.,* 2016), haplotypic duplication was identified and removed with purge_dups ( Guan *et al.,* 2020) and a first round of scaffolding carried out with 10X Genomics read clouds using scaff10x ( https://github.com/wtsi-hpag/Scaff10X). Scaffolding with Hi-C data ( Rao *et al.,* 2014) was carried out with 3D-DNA ( Dudchenko *et al.,* 2017), followed by manual curation with Juicebox Assembly Tools ( Dudchenko *et al.,* 2018; Durand *et al.,* 2016; Robinson *et al.,* 2018) and visualisation in HiGlass ( Kerpedjiev *et al.,* 2018). The Hi-C scaffolded assembly was polished with arrow using the PacBio data, then polished with the 10X Genomics Illumina data by aligning to the assembly with longranger align, calling variants with freebayes ( Garrison & Marth, 2012) and applying homozygous non-reference edits using bcftools consensus ( https://github.com/VGP/vgp-assembly/tree/master/pipeline/freebayes-polish). Two rounds of the Illumina polishing were applied. The assembly was checked for contamination and corrected using the gEVAL system ( Chow *et al.,* 2016). We removed two low-coverage scaffolds that were likely to have derived from the ribosomal DNA cistron of a *Sarcocystis* species (most similar to *Sarcocystis lutrae*). The genome was analysed within the BlobToolKit environment ( Challis *et al.,* 2019).

## Data availability

European Nucleotide Archive: Lutra lutra (Eurasian otter) genome assembly, mLutLut1. BioProject accession number PRJEB35340; https://www.ebi.ac.uk/ena/data/view/PRJEB35340.

The genome sequence is released openly for reuse. The *L. lutra* genome sequencing initiative is part of the Wellcome Sanger Institute’s “25 genomes for 25 years” project ^3^. It is also part of the Vertebrate Genome Project (VGP) ^4^ ordinal references programme, the DNA Zoo Project ^5^ and the Darwin Tree of Life (DToL) project ^6^. The specimen has been preserved in ethanol and deposited with the Natural History Museum, London under registration number NHMUK ZD 2019.215 where it will remain accessible to the research community for posterity. All raw data and the assembly have been deposited in the ENA. The genome will be annotated and presented through the Ensembl pipeline at the European Bioinformatics Institute. Raw data and assembly accession identifiers are reported in Table 1.

## Footnotes

^1^Vincent Wildlife Trust https://www.vincentwildlife.ie/species/otter

^2^National Biodiversity network Atlas https://species.nbnatlas.org/species/NBNSYS0000005133#overview

^3^ https://www.sanger.ac.uk/science/collaboration/25-genomes-25-years

^4^ https://vertebrategenomesproject.org/

^5^ https://www.dnazoo.org/

^6^ https://www.darwintreeoflife.org/
