# Supplementary material for: The genome sequence of the Eurasian river otter, Lutra lutra Linnaeus 1758
Source: Wellcome Open Res. 2020 Feb 19;5:33. [Version 1] doi: 10.12688/wellcomeopenres.15722.1 (PMC7097881; doi:10.12688/wellcomeopenres.15722.1)
Supplement: Peer review history

# Peer review: Reviewer response for version 1

**DOI:** 10.21956/wellcomeopenres.17235.r37966

**Published:** 2020-03-17

**Authors:** Frank Panitz

**Affiliations:** 1 Department of Molecular Biology and Genetics, Aarhus University, Aarhus, Denmark

The manuscript describes the *de novo* genome assembly of the Eurasian river otter ( *Lutra lutra*). The authors combine the latest state-of-the art sequencing methods, PacificBioscience and 10X Genomics, to generate primary contigs which are then assembled into 43 scaffolds, having a high N50 value of 149 Mb, using Hi-C data. In addition, 92.7% of the total assembly are assigned to chromosomal pseudomolecules, representing 18 autosomes as well as X and Y sex chromosomes. Together with a high level of completeness as analysed by BUSCO quality assessment, this *de novo* assembly provides a high-quality draft genome sequence.

 The genome resources generated in this project will be instrumental to investigate the genetic structure, genetic diversity and phylogeny of Eurasian otters. As monitoring tools are urgently needed for development and evaluation of conservation efforts the genome sequence will provide genetic markers to be used in conservation genetic studies.

 The manuscript is concise, data is presented comprehensively and the figures are linked to interactive versions

 Recommendation:

 The Busco software used for genome quality assessment should to be included in Table 3.

I confirm that I have read this submission and believe that I have an appropriate level of expertise to confirm that it is of an acceptable scientific standard.

# Peer review: Reviewer response for version 1

**DOI:** 10.21956/wellcomeopenres.17235.r37967

**Published:** 2020-03-05

**Authors:** Yibo Hu

**Affiliations:** 1 CAS Key Laboratory of Animal Ecology and Conservation Biology, Institute of Zoology, Chinese Academy of Sciences, Beijing, China

This manuscript described the detailed information about the chromosome-level *de novo* genome assembly of the Eurasian river otter. They used PacBio SMRT and 10X Genomics sequencing techniques to construct the scaffolds of the genome. These two techniques are widely popular sequencing methods that are good at constructing long scaffolds. They assembled as low as 43 scaffolds. Furthermore, they used Hi-C technique to assign the scaffolds on chromosomes. As a result, 92.7% of the assembly sequences were assigned to 20 chromosomal pseudomolecules. The levels of contig N50 and scaffold N50, and BUSCO genome assembly assessment showed that the genome assembly is of high-quality.

 The Eurasian river otter is listed as Near Threatened under the IUCN red list, and some populations have been decreasing. So, it is important to know the genetic diversity, genetic structure and adaptive evolution mechanisms of Eurasian river otters. The chromosome-level genome assembly will help answer the above questions, which has important conservation implications for this species.

 The report about the genome assembly was detailed, and the manuscript written well. I just have two comments.

1. I suggest to add a photo of Eurasian river otter to show this species to readers.
2. Table 2: it is better to add the number of scaffolds assigned for each chromosome.  

I confirm that I have read this submission and believe that I have an appropriate level of expertise to confirm that it is of an acceptable scientific standard.
